# Supplementary material for: Near-field terahertz probes with room-temperature nanodetectors for subwavelength resolution imaging
Source: Sci Rep. 2017 Mar 13;7:44240. doi: 10.1038/srep44240 (PMC5347152; doi:10.1038/srep44240)
Supplement: Supplementary File [file srep44240-s1.pdf]

Supplementary Information

Near-field terahertz probes with room-temperature nanodetectors for subwavelength resolution imaging

Oleg Mitrofanov,[1] Leonardo Viti,[2] Enrico Dardanis,[2] Maria Caterina Giordano,[2] Daniele Ercolani,[2] Antonio Politano,[3] Lucia Sorba,[2] and Miriam S. Vitiello [2*]

[1] *University College London, Electronic and Electrical Engineering, London, WC1E 7JE, UK*

[2] *NEST, Istituto Nanoscienze – CNR and Scuola Normale Superiore, Piazza San Silvestro 12, Pisa, I-56127*

[3] *Università degli Studi della Calabria, Dipartimento di Fisica, via Ponte Bucci, 87036 Rende (CS), Italy*

1.1 Determination of the Seebeck coefficient for sample A

The Seebeck coefficient can be retrieved using the Mott formula:

$$S_b = -\frac{\pi^2 k^2 T}{3e} \cdot \frac{1}{\sigma} \cdot \left.\frac{d\sigma}{d\varepsilon}\right|_{\varepsilon=E_F} \qquad \text{(Eq. S1)}$$

where k is the Boltzmann constant, T is the temperature, σ is the channel conductivity, ε is the energy and E_F is the Fermi energy. The electrical behavior of sample A shows that the device operates in the linear transport regime in the range of gate voltages: V_G = -0.35 V - 0.25 V, as shown in the transconductance curve (Figure S1a). In this regime, the carrier mobility is constant and Eq. S1) can be re-written as [36]:

$$S_b = -\frac{\pi^2 k^2 T}{3C_{gw}} \cdot \left(\frac{1}{\sigma} \cdot \frac{d\sigma}{dV_G}\right) \frac{dn}{dE_F} \qquad \text{(Eq. S2)}$$

Where C_{gw} is the capacitance between the nanowire and the gate electrode, normalized to the gated volume, and n the carrier density. The derivative dn/dE_F can be estimated from the electron density in the InAs nanowire:

$$n(E_F) = \int_0^\infty D(E) f(E) dE \qquad \text{(Eq. S3)}$$

Where $D(E)$ is the 3-dimensional density of states (this approximation is valid for nanowires with radius larger than 20 nm) and $f(E)$ is the Fermi-Dirac Distribution:

$$D(E) = \frac{1}{2\pi^2}\left(\frac{2m^*}{\hbar^2}\right)^{\frac{3}{2}}\sqrt{E} \qquad \text{(Eq. S4)}$$

$$f(E) = \frac{1}{\exp\left(\frac{E-E_F}{kT}\right)+1} \qquad \text{(Eq. S5)}$$

where $E = 0$ corresponds to the bottom of the conduction band, and $m^* = 0.023\ m_0$ is the electron effective mass in InAs, Equation (S3) allows us to retrieve the trends of Fig. S1,b and Fig. S1,c. Under the assumption of constant mobility (μ = 500 cm^2/Vs across the linear region, as estimated from the measurement of transcoductance), the channel conductivity σ and the electron density n are related via the following equations:

$$\sigma(V_G) = n(V_G)e\mu, \quad \sigma(E_F) = n(E_F)e\mu, \qquad \text{(Eq. S6)}$$

Equations S6 allow us to define the function $E_F(V_G)$ in the linear region of the FET. Furthermore, our experiments indicate that by changing V_G, the carrier density varies between 7×10^{17} cm^{-3} (V_G = -0.35 V) and 10^{18} cm^{-3} (V_G = 0.25 V). In this bias interval (green shaded area in Figure S1b,c) $dn(E_F)/dE_F$ can be approximated as a linear function and the Seebeck coefficient (S_b) can be then evaluated (Figure S1,d). The S_b values are is agreement with previously reported values on similar structures [37].

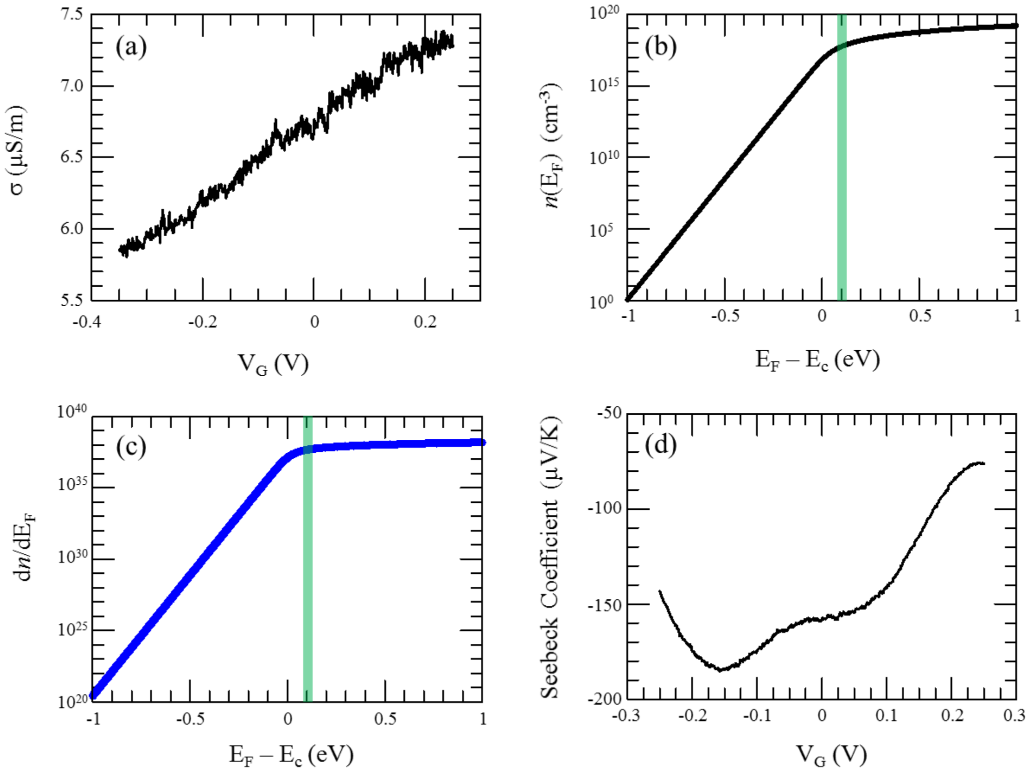

Figure S1. Sample A (a) Channel conductivity as a function of V_G measured in the range from -0.35 V to 0.25 V; (b) Carrier density as a function of the Fermi level, calculated from equation (S3); (c) first derivative of the carrier density. The green shaded area represents the energy span covered by changing the gate voltage from -0.35 V to 0.25 V, as determined from equation S6. (d) Computed Seebeck coefficient S_b as a function of V_G.

1.2 Determination of the Seebeck coefficient for sample B

While analyzing the transport behavior of sample B, two different transport regimes can be identified (Figure S2a): region I (blue shaded linear region in the logarithmic plot of Fig. S2b) corresponding to the subthreshold regime (V_G = 0.5 V-1 V); region II corresponding to the linear transport regime (V_G = -1.5 V - 0.5 V). To extract the Seebeck coefficient S (blue curve in Fig. S2d) at V_G > 0.5 V we investigated the subthreshold swing $\frac{edV_G}{dE_f}$, which is ≈ β [38]:

$$S_b = -\frac{\pi^2 k^2 T}{3e} \cdot \left(\frac{1}{\sigma} \cdot \frac{d\sigma}{dV_G}\right) \frac{dV_G}{dE_F} \qquad \text{(Eq. S7)}$$

Here β can be extracted from the linear fit of the logarithm of the current vs V_G in the subthreshold region. Conversely, in region II (gray region in Figure S2b) we can make the same assumptions used for the calculation of the Seebeck coefficient for sample A, after substituting in Eq. S2, C_{gw} with the gate to channel capacitance per unit area C_{gc}, and by retrieving the density of carriers per unit area via the equation [39]:

$$n(E_F) = \sum_j \frac{m^j_{DOS} k_B T}{\pi \hbar^2} \ln\left[1 + \exp\left(\frac{E_f - E^j{}_c}{k_B T}\right)\right] \qquad \text{(Eq. S8)}$$

The energies $E^j{}_c$ here indicate the subbands arising in a BP flake of finite thickness as an effect of the confinement in the out-of-plane *z* direction. The $n(E_F)$ plot calculated via Eq. S8 is shown in Fig. S2c. Combining Eqs. S6 and S8 we derived dn/dE_f and then S_b (red curve in Figure S2c) via Eq. S2.

The overall Seebeck coefficient of sample B, plotted in Fig. 5b of the main text, can be then achieved combined the two curves discussed above and correspond to the red curve (Fig. S2d) below V_G = 0 V and to the blue curve (Fig. S2d) above V_G = 0 V. The two curves well combine at V_G = 0 V, if we set the mobility of the BP flake at 900 cm^2/Vs across the linear region, a value which is expected for BP flakes, having thickness comparable with the present experimental case.

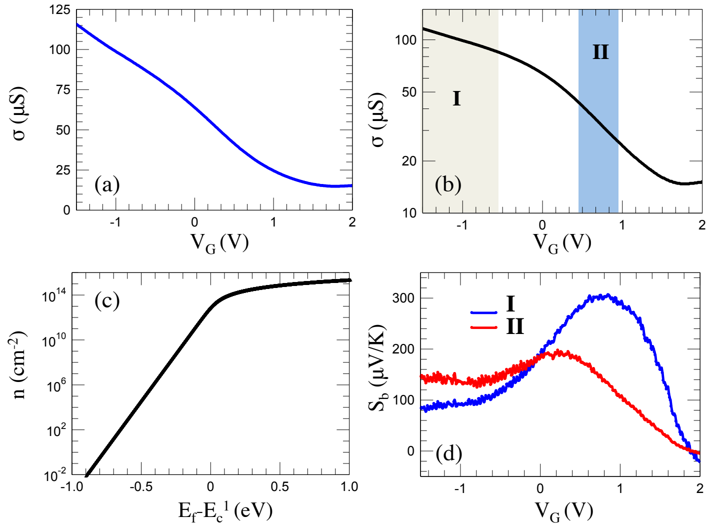

Figure S2. Sample B. (a) Channel conductivity as a function of V_G. (b) Logarithmic plot of the conductivity; region I and II t are highlighted via shadow areas (c) Surface carrier density for a 38 nm thick BP flake as computed with Eq. S8. (d) Computed Seebeck coefficient as a function of V_G.

2.1 Phase-sensitive interferometric setup

The interferometric setup exploited for phase sensitive experiments is depicted in Figure 4 in the main text. A transparent chip carrier has been used, so that the detector can be simultaneously excited by two optical beams (Figure 4 right, upper panel), one impinging on the aperture (I_1, signal) and the other on the back-side (I_{REF}, reference) through the Si substrate. The QCL beam is first collimated by a Picarin lens having focal length of 3 cm and then hits a double-side polished intrinsic silicon wafer which acts as beam-splitter (BS). The estimated BP reflectance is 65%, whereas its transmittance is 35% (assuming the material lossless). This sets $I_{REF} \sim 2I_1$. After reflection on the BS, the reference beam is sent through a delay stage built with a set of four, gold-coated flat mirrors (M1, M2, M3, M4). Then it's focused on the back-side of the near-field probe by an off-axis parabolic mirror (P1) having a focal length f_1 = 5 cm. Simultaneously, the signal beam, transmitted by the BS, hits a flat mirror (M5) and is then focused on the probe aperture by an off-axis parabolic mirror (P2) having focal length f_2 = 2.5 cm.

3.1 Evaluation of the spatial resolution

The spatial resolution of a 20 μm aperture near-field probe has been assessed by mapping the THz intensity distribution formed at the tips of two metallic needles separated by ~ 10 μm, brought in close proximity (~ 2 μm) to the aperture (see inset of Figure S3) and placed in the focus of the beam [40]. The spatial size of the confined THz field is determined by the distance between the two tips and is narrower than the aperture size. Thus, the signal profile obtained by moving the detector in front of the needles shown in Figure S3 is the near-field detector response to a point-like source. The full width at half maximum (FWHM) of the intensity profile then represents the spatial resolution of the ~ 17 μm aperture detector.

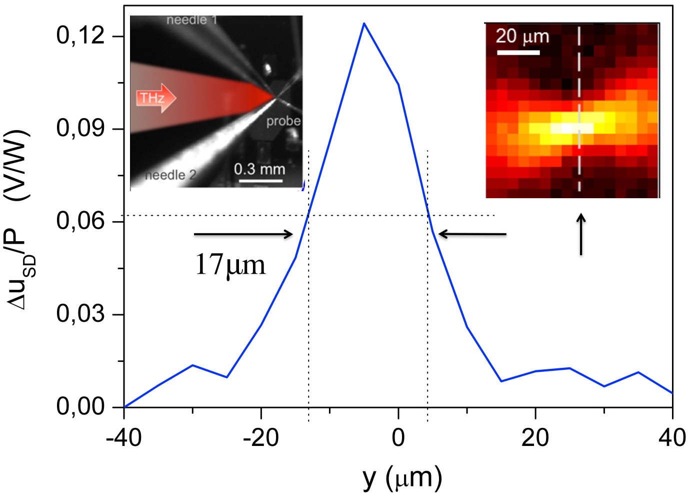

Figure S3. Profile of the detected photovoltage Δu_{SD}, normalized to the total input power, for THz illumination in presence of two sharp needles in the vicinity of the aperture (inset). The detected signal reveals a spatial resolution of about 17 μm evaluated as the FWHM of the peak. The inset shows 2-dymensional color plot of Δu_{SD}.

REFERENCES

[36] Low, T., Engel, M., Steiner, M., and Avouris, P. Origin of photoresponse in black phosphorus phototransistors. *Phys. Rev. B* **90**, 081408 (2014).

[37] Roddaro, S., Ercolani, D., Safeen, M. A., Suomalainen, S., Rossella, F., Giazotto, F., Sorba, L. and Beltram, F. Giant Thermovoltage in Single InAs Nanowire Field-Effect Transistors. *Nano Lett.* **13**, 3638-3642 (2013).

[38] Viti, L., Hu, J., Coquillat, D., Knap, W., Tredicucci, A., Politano, A., & Vitiello, M. S., Black Phosphorus Terahertz Photodetectors *Adv. Mat. 27*(37), 5567-5572. (2015)

[39] Low, T., Rodin, A. S., Carvalho, A., Jiang, Y., Wang, H., Xia, F., Castro Neto, A. H. Tunable optical properties of multilayer black phosphorus thin films. *Phys. Rev. B* **90**, 075434 (2014)

[40] Mitrofanov, O., Renaud, C.C., Seeds, A.J., Terahertz probe for spectroscopy of sub-wavelength objects. *Optics express* 20, 6197-6202 (2012)..
